# The effect of intensity on metabolic and ventilatory responses to steady-state exercise in women across the adult lifecycle

**DOI:** 10.1007/s00421-025-05981-1

**Published:** 2025-09-19

**Authors:** Catherine A. Rattley, Paul Ansdell, Matthew Armstrong, Malika Felton, Susan Dewhurst, Karen Yendole, Rebecca A. Neal

**Affiliations:** 1 Division of Surgery and Interventional Science, Institute of Sport, Exercise and Health, University College London, London, UK; 2 Department of Sport, Exercise and Rehabilitation, Faculty of Health and Life Sciences, Northumbria University, Newcastle-Upon-Tyne, UK; 3 Department of Sport and Exercise Sciences, Faculty of Social Sciences and Health, Durham University, Durham, UK; 4 Department of Rehabilitation and Sport Sciences, Faculty of Health and Social Science, Bournemouth University, Bournemouth, UK; 5 Department of Life and Environmental Sciences, Faculty of Science and Technology, Bournemouth University, Bournemouth, UK

**Keywords:** exercise physiology, menopause, cardiopulmonary

## Abstract

**Aim:**

This study aimed to investigate the differences in metabolism and ventilation between women before, during, and after menopause during rest and to varying steady-state exercise intensities.

**Method:**

74 female participants (18–60 years; premenopausal [PRE], perimenopausal [PERI], and postmenopausal [POST]) completed four laboratory visits; one maximal exercise test, resting data collection, and body composition assessment followed by three steady-state submaximal exercise tests at 40% (low), 60% (moderate), and 80% (high) V̇O_2peak_ in a randomised order with subgroup analysis for hormonal contraceptive or hormone therapy use.

**Results:**

There was an effect of menopause stage on exercise energy expenditure but no interaction effect with intensity. There were no differences in substrate utilisation or ventilation across any of the exercise intensities. Subgroup analysis revealed that HT and HC use did not impact EE, substrate oxidation, or ventilation.

**Conclusions:**

Menopause stage is influential upon exercise energy expenditure but more research in perimenopause is required to confirm the effect, future studies should explore the broader implications of the menopausal transition on exercise physiology.

**Supplementary Information:**

The online version contains supplementary material available at 10.1007/s00421-025-05981-1.

## Introduction

During the menopause transition, fat gain can increase two-to-fourfold (Greendale et al. 2019), particularly intra-abdominal fat (Toth et al. 2000), and lean body mass can also decrease (Davis et al. 2012; Greendale et al. 2019). These changes stabilise in postmenopause (Greendale et al. 2019). Increases in body fat may result from reductions in resting and exercise energy expenditure (EE) as a result of declining oestrogen and progesterone (Lovejoy et al. 2008; Abildgaard et al. 2013; Melanson et al. 2015) in addition to the effects of ageing (Müller et al. 2002). Crucially, reductions in EE can result in an increased number of risk factors, including increased waist circumference, elevated triglycerides, elevated fasting glucose, and elevated blood pressure (Pu et al. 2017), which may contribute to increased risk of cardiovascular disease and type 2 diabetes (Marlatt et al. 2022).

Oestrogen supplementation has been demonstrated to increase reliance on fat oxidation (FATox) in males (Hamadeh et al. 2005) and in rats (Kendrick et al. 1987). This is suggested to be due to increased plasma free fatty acid availability, increased muscle triacylglycerol content (Hamadeh et al. 2005), increased skeletal muscle uptake of glucose and suppression of gluconeogenesis limiting blood glucose availability (Oosthuyse et al. 2022), and delayed onset of glycogen oxidation (Kendrick et al. 1987). Acute fluctuations in sex hormones throughout the menstrual cycle have not been consistently demonstrated to alter substrate oxidation at rest or in submaximal exercise (Horton et al. 2002; Williams et al. 2023) nor the ability to efficiently shift between fuel sources in response to changing energy demands, termed metabolic flexibility (Olenick et al. 2023). However, the size of the ratio of oestrogen to progesterone (Hackney et al. 2022; Oosthuyse et al. 2022), as well as diet composition or fasted state prior to exercise (Oosthuyse et al. 2022), may explain this inconsistency.

Whilst the pattern of shifting from FATox to carbohydrate oxidation (CHOox) with increasing exercise duration remains in postmenopausal females (Johnson et al. 2002), it is suggested that metabolic flexibility decreases in perimenopause and remains this way in postmenopause (Lovejoy et al. 2008; Gould et al. 2022), which may lead to impaired energy homeostasis (Muoio 2014). Menopause is suggested to decrease the hormonally driven reliance on fat (Isacco et al. 2012), such that during low-intensity exercise, postmenopausal females have evidenced a lower FATox than premenopausal and perimenopausal females (Melanson et al. 2015; Gould et al. 2022) demonstrating a reduced metabolic efficiency. Additionally, an inability to oxidise fat has been labelled as an important contributor to obesity and type 2 diabetes (Achten and Jeukendrup 2004), subsequently identifying this point of shift towards lesser FATox and altered EE during menopause may enable intervention to reduce visceral fat mass and retain metabolic flexibility (Gould et al. 2022).

Similarly, ventilation may also be hormonally regulated, specifically by progesterone, a ventilatory stimulant (Behan and Kinkead 2011). Resting minute ventilation (V̇E) has been demonstrated to be elevated in the luteal phase of the menstrual cycle (Dombovy et al. 1987; Das 1998; Slatkovska et al. 2006; MacNutt et al. 2012), resulting from increased concentrations of progesterone in this phase compared to the follicular phase (León-Velarde et al. 2001). Hence, in the absence of progesterone, ventilation may decrease. Current studies on resting and peak V̇E report no differences between the premenopause and postmenopause (Mercuro et al. 2006; Preston et al. 2009; Rael et al. 2021); however, ventilation in submaximal exercise is unexplored.

Study of the physiological response to steady-state exercise during various stages of the female lifecycle, with considerations for the interlinked cardiopulmonary and metabolic physiology, will enable a greater understanding of the impact of sex hormone declines on females in midlife. It is hypothesised that postmenopausal females will exhibit lower EE, FATox, and V̇E compared to premenopausal and perimenopausal females due to chronically low levels of sex hormones oestrogen and progesterone. For the first time, this study aimed to examine the effect of perimenopause and postmenopause on ventilatory and metabolic responses at varying steady-state exercise intensities.

## Method

### Participants

**Fig. 1:**
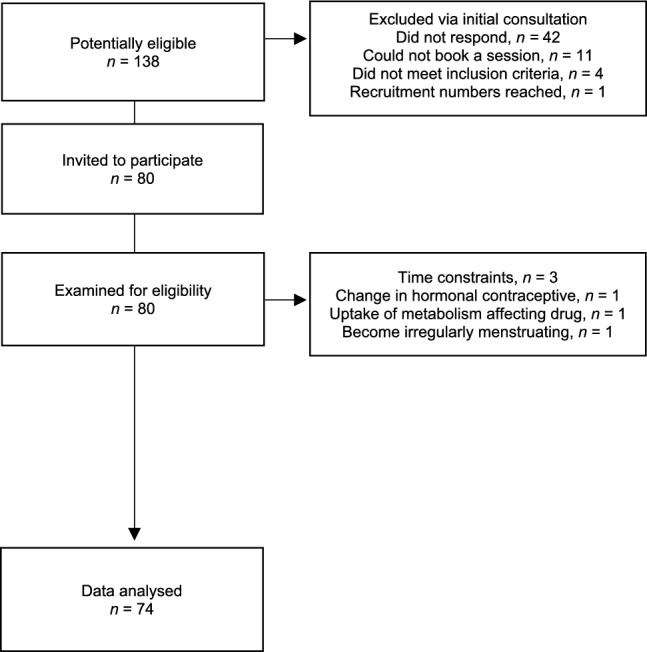
Participant flow throughout the study

An a priori power calculation for a repeated between factors analysis of variance (ANOVA), based on an effect size of 0.40 for respiratory quotient (RQ) from Gould et al. (2022), determined that a total of 69 participants would be statistically powered to 95% (G*Power 3.1.9.7, Heinrich-Heine-Universität Düsseldorf; Faul et al. 2007). Power calculation details are available in supplementary materials. Accounting for a drop out of approximately 20%, 80 female participants (reproductive, perimenopausal, postmenopausal) were recruited to take part in this study aiming for 26 in each group. Six participants did not complete testing: three due to time constraints, one due to change in hormonal contraceptive, one due to uptake of metabolism-affecting drug, and one became irregularly menstruating during study process. Therefore, 74 participants completed the study (Fig. 1).

Menopause status of each individual was defined as follows (Harlow et al. 2012; Sarri et al. 2015; Ambikairajah et al. 2022):

- Premenopause (PRE): between the ages of 18 and 45 with menstrual regularity as defined by a cycle of 21–35 days, or not symptomatic if using hormonal contraceptive.
- Perimenopause (PERI): persistent > 7 days difference in length of consecutive cycles or interval of amenorrhea of > 60 days **and/or** vasomotor, musculoskeletal or mood symptoms if using hormonal contraceptive or hormone therapy.
- Postmenopause (POST): the period after 12 consecutive months of amenorrhea.

#### Procedures

On attendance to the laboratory, Participants provided informed consent and were screened for normotensive blood pressure. Participants undertook four visits to the laboratory. Each participant was required to perform one maximal exercise test, and three submaximal exercise tests in a randomised counterbalanced order. Upon arrival to the initial study visit, participants completed a body composition assessment, and then had a resting supine venous blood sample taken followed by a 10-min resting energy expenditure assessment seated. Finally, a maximal exercise assessment was conducted. In subsequent visits, body composition was assessed followed by the required steady-state exercise test.

Naturally menstruating premenopausal and perimenopausal participants completed screening and all exercise testing in the early-to-midfollicular phase within 7 days of starting a menstrual bleed; to more closely align with the low oestradiol state in postmenopause. Based on recommendations from Schmalenberger et al. (2021) on study of the menstrual cycle, studying between day 1 and day 7 ensures that even participants with shorter cycles (21 days) are studied prior to ovulation. This ensures all participants are in the early-to-midfollicular phase of their menstrual cycle.

Therefore, experimental sessions were completed at least 1 month apart. Those who were using hormonal contraceptive, were perimenopausal and had not had a bleed for over 60 days, or were postmenopausal were tested at their earliest convenience. For these participants, experimental sessions were at least 1 week apart. Participants attended at the same part of the day to account for diurnal variability, either morning, afternoon, or evening. Participants were advised not to exercise in the 48 h prior to maximal exercise protocol and in the 24 h prior to each steady-state exercise visit. Participants were also advised to eat over 2 h before the test.

#### Anthropometrics

Participants then had measurements of anthropometrics taken by stadiometer (217, SECA, Hamburg, Germany) and body mass scales (803, SECA, Hamburg, Germany) followed by body composition measurement using bioelectrical impedance scales (InBody770, InBody Ltd, Seoul, South Korea).

#### Blood hormone measurements

Laid supine, a 6 mL venous blood sample was collected into an EDTA vacutainer, which was centrifuged for 10 min at 300 rpm at 4 °C, and the plasma removed into duplicate 1.5 mL Eppendorfs. The plasma was stored at -80 °C and tested within a maximum of 12 months. The plasma samples were assessed using Enzyme Linked Immunosorbent Assays (ELISA) (Human Estradiol ELISA Kit, ABCAM, Cambridge, UK; Human Progesterone ELISA Kit, ABCAM, Cambridge, UK) and analysed via plate reader (ELX800 Microplate reader, BioTek, Vermont, United States). Minimum detectable plasma concentrations were 8.68 pg mL^−1^ for oestradiol and 0.05 ng mL^−1^ for progesterone. Intra-assay coefficient of variation was 23.7% for oestradiol and 14.9% for progesterone.

#### Resting energy expenditure

Following this 5 minutes in supine position, participants had seated resting data collected for 10 min wearing a mask connected to a metabolic cart (K5, COSMED, Rome, Italy) measuring expired gases.

#### Maximal exercise test

Participants completed the maximal exercise test (V̇O_2peak_) on a cycle ergometer (Lode Excalibur Sport, Lode BV, Groningen, The Netherlands). The test began with a 3-min period of cycling increasing up to 50 Watts (W) for the warm-up after which power output continually increased until the participant could no longer continue despite strong verbal encouragement. Adapted from the protocol by Pollock et al. (2018), whereby the rate of increase was 1 W every 3–5 s, participants cycled at a self-selected cadence over 70 rpm and, dependent on self-reported activity level in a screening questionnaire, resistance was increased by one W every 5 s (for high activity level) or 7 s (for moderate activity level). Pollock et al. (2018) devised this protocol in masters’ athletes, and therefore, this decreased rate of increase was required on the involvement of below elite populations and determined based on pilot testing. Directly before the maximal test, a fingertip blood sample was taken for a resting lactate measurement (Biosen C-Line, EKF diagnostics, Barleben, Germany) and a final lactate measurement immediately after completion of the test. A rating of perceived exertion was given every 2 min.

#### Submaximal exercise tests

The submaximal tests consisted of 30 min at 40%, 60%, or 80% of V̇O_2peak_ (McCole et al. 1999; Johnson et al. 2002) to mimic ecologically valid doses of cardiovascular exercise, i.e., exercise intensities and dosages that reflect real-world physical activity practises (Dalleck et al. 2009; Hansen et al. 2018). Participants cycled at a self-selected cadence and completed a warm-up of 2 min at 0 W at which point resistance increased until the participant reached the prescribed V̇O_2_ followed by 30 min at the determined intensity. Power output (W) was adjusted in 1–5 Watt increments as required to maintain intensity by continuous monitoring of V̇O_2_ to ensure the desired metabolic stimulus throughout the test (Teso et al. 2022).

For all exercise testing, expired gases were measured by metabolic cart (K5, COSMED, Rome, Italy) which was calibrated in line with the manufacturer’s instructions. Heart rate data were collected by chest heart rate monitor (Polar H10, Polar, Kempele, Finland). Variables extracted from expired gases included V̇O_2_, V̇CO_2_, respiratory frequency (RF), V̇E, tidal volume (TV), and RQ.

#### Physical activity diary

After the first visit, participants were requested to fill out a 3-week physical activity diary to confirm activity level by leisure time metabolic equivalent (METS) minutes per week utilising the METS compendium (Ainsworth et al. 2011) to assign value to exercise types. Leisure time METS were calculated as the MET for the activity multiplied by duration in minutes. Nine participants (three PRE, four PERI and two POST) failed to provide physical activity diaries.

### Data analysis

EE, and CHOox and FATox rates were calculated using the following equations from Weir (1949) and Jeukendrup and Wallis (2005), respectively. Negative values were interpreted as zero. EE and FATox were also normalised to FFM.

Energy expenditure across all intensities:

$${\mathrm{Energy}} \;{\mathrm{expenditure}}\left( {{\mathrm{kcal}}\;\min^{ - 1} } \right) = \left( {3.941 \times {\mathrm{VO}}_{2} \left( {L\;\min^{ - 1} } \right)} \right) - \left( {1.106 \times {\mathrm{VCO}}_{2} \left( {L\;\min^{ - 1} } \right)} \right).$$

Fat oxidation across all intensities:

$${\mathrm{Fat}} \cdot {\mathrm{oxidation}}\left( {g\;\min^{ - 1} } \right) = \left( {1.695 \times {\mathrm{VO}}_{2} \left( {L\;\min^{ - 1} } \right)} \right) - \left( {1.701 \times {\mathrm{VCO}}_{2} \left( {L\;\min^{ - 1} } \right)} \right).$$

Carbohydrate oxidation at 40% V̇O_2 max_:

$${\mathrm{Carbohydrate}} \cdot {\mathrm{oxidation}}\left( {g\;\min^{ - 1} } \right) = \left( {4.344 \times {\mathrm{VCO}}_{2} \left( {L\;\min^{ - 1} } \right)} \right) - \left( {3.061 \times {\mathrm{VO}}_{2} \left( {L\;\min^{ - 1} } \right)} \right).$$

Carbohydrate oxidation at 60% and 80% of V̇O_2 max_:

$${\mathrm{Carbohydrate}}\;{\mathrm{oxidation}}\left( {g\;\min^{ - 1} } \right) = \left( {4.210 \times {\mathrm{VCO}}_{2} \left( {L\;\min^{ - 1} } \right)} \right) - \left( {2.962 \times {\mathrm{VO}}_{2} \left( {L\;\min^{ - 1} } \right)} \right).$$

Exercise intensity was calculated post hoc as a percentage of V̇O_2_ reserve. V̇O_2_reserve was calculated by deducted resting V̇O_2_ from maximal V̇O_2_. Then, percentage of V̇O_2_ reserve

$${\mathrm{VO}}_{2} {\mathrm{reserve}} \left( \% \right) = \frac{{{\mathrm{Sub}}\;{\mathrm{maximal}} \;{\mathrm{VO}}_{2} - {\mathrm{Resting}} \;{\mathrm{VO}}_{2} }}{{{\mathrm{VO}}_{2} \max - {\mathrm{Resting}}\; {\mathrm{VO}}_{2} }} \times 100.$$

Predicted V̇O_2max_ was calculated using the FRIEND equation (Myers et al. 2017)

$${\mathrm{VO}}_{2\max } \left( {{\mathrm{ml}}\;{\mathrm{kg}}\;\min } \right) = 79.9 - \left( {0.39 \times {\mathrm{age}}} \right) - \left( {13.7 \times 1} \right) - \left( {0.127 \times {\mathrm{weight}} \left[ {{\mathrm{lbs}}} \right] } \right).$$

All data are presented as mean and standard deviation (SD). Breath by breath data were smoothed over 15 s and were then averaged over five-minute intervals for all variables and then a mean for entire exercise bout calculated. V̇O₂peak was defined as the highest smoothed 15-s oxygen uptake value obtained during the ramp maximal test. Due to 17 participants terminating the 80%V̇O_2max_ condition early at 20 min and six participants terminating at 25 min, data are presented for 20 min of exercise at 80% V̇O_2peak_.

#### Statistical analysis

All data were assessed for normality using Shapiro–Wilk test for normality. For demographic data, where data were normally distributed, one-way ANOVAs were utilised with a Tukey’s multiple comparisons test. Where data were non-normally distributed a Kruskal–Wallis test with Dunn’s multiple comparisons was used. Where data were normally distributed but with unequal variances identified by Bartlett’s test, a Welch’s ANOVA was used in place with Dunnett’s T3 multiple comparisons test. For exercise data, a two-way ANOVA (intensity x group) was utilised. Statistical analyses was conducted using GraphPad Prism (Version 9.5.0, GraphPad Software, Boston, Massachusetts USA). Significance was indicated at a *p* value of < 0.05.

## Results

All groups were significantly different in age (*p* > 0.001). The PRE group ranged from 18 to 45 years old, PERI 41 to 56 years old, and POST 48 to 60 years old. POST had lower muscle mass and FFM than PRE (*p* = 0.001, *p* = 0.005, respectively) and PERI (*p* = 0.003, *p* = 0.006, respectively). POST also had a higher body fat percentage than PRE (*p* = 0.019). See Table 1. PERI evidenced a higher oestrogen than POST (*p* = 0.012).

**Table 1:** Mean and standard deviation for participant characteristics and for resting data averaged for 10 min

	PRE (*n* = 35)	PERI (*n* = 19)	POST(*n* = 20)	*p*
Age (years)	32 ± 7^*†‡*^	47 ± 4*^‡^	55 ± 3*^†^	** < 0.001**
Height (cm)	168.9 ± 6.0^*‡*^	169.0 ± 5.4^*‡*^	164.7 ± 4.2*^†^	**0.015**
Weight (kg)	69.0 ± 9.8	71.4 ± 10.1	69.0 ± 11.7	0.689
BMI (kg m^−2)^	24.1 ± 3.3	25.0 ± 3.4	25.5 ± 4.6	0.380
Body fat (%)	25.3 ± 8.2^*‡*^	26.6 ± 6.3	31.4 ± 8.5*	**0.024**
Body fat mass (kg)	20.2 ± 7.1	19.4 ± 6.9	22.5 ± 10.2	0.447
Muscle mass (kg)	28.3 ± 2.9^*‡*^	28.5 ± 3.0^*‡*^	25.5 ± 1.9*^†^	**0.001**
Fat-free mass (kg)	50.7 ± 4.8^*‡*^	51.2 ± 5.4^*‡*^	46.5 ± 3.4*^†^	**0.002**
Oestradiol (pg ml)	29.1 ± 32.4	54.3 ± 65.5^*‡*^	18.3 ± 21.9^*†*^	0.016
Progesterone (ng ml)	0.72 ± 0.75	1.04 ± 1.80	1.05 ± 2.3	0.074
Metabolic equivalent minutes per week	2676.9 ± 1121.3^*†*^	1935.9 ± 671.2*	2062.5 ± 1054.5	**0.018**
V̇O_2peak_ (mL kg^−1^ min^−1^)	39.5 ± 7.9	37.2 ± 6.2	35.0 ± 6.4	0.081
Predicted V̇O_2max_ (mL kg^−1^ min^−1^)	34.2 ± 7.7^*†‡*^	27.8 ± 3.4*	25.3 ± 2.5*	** < 0.001**
Difference between measured V̇O_2peak_ and predicted V̇O_2max_ (%)	16% ± 20%^*†‡*^	34% ± 18%*	38% ± 10%*	** < 0.001**
Difference between measured V̇O_2peak_ and predicted V̇O_2max_ (mL kg^−1^ min^−1^)	5.2 ± 6.77^*†‡*^	9.4 ± 5.0*	9.6 ± 5.5*	**0.012**
Resting				
V̇O_2_ (mL kg^−1^ min^−1^)	5.3 ± 1.3^*‡*^	4.8 ± 1.1	4.5 ± 1.0*	**0.048**
Energy expenditure (kcal min^−1^)	1.7 ± 0.4	1.6 ± 0.3	1.5 ± 0.3*	**0.003**
Fat oxidation (g min^−1^)	0.10 ± 0.05	0.10 ± 0.05	0.10 ± 0.03	0.965
Carbohydrate oxidation (g min^−1^)	0.22 ± 0.13	0.19 ± 0.11	0.14 ± 0.07*	**0.025**
Respiratory frequency (1 min^−1^)	16.7 ± 2.0	16.0 ± 2.7	15.4 ± 2.5	0.111
Tidal volume (L)	0.7 ± 0.2	0.7 ± 0.2	0.6 ± 0.1	0.069
Minute ventilation (L min^−1^	11.0 ± 2.4	10.2 ± 2.0	9.0 ± 1.8*	**0.001**
Respiratory quotient	0.84 ± 0.06	0.83 ± 0.07	0.81 ± 0.05	0.142
Ventilatory equivalents (V̇E/V̇CO_2_)	32.5 ± 2.3	32.6 ± 3.3	32.6 ± 2.7	0.954
Ventilatory equivalents (V̇E/V̇O_2_)	27.4 ± 1.7	26.9 ± 2.2	26.3 ± 2.2	0.263
P_ET_CO_2_	32.8 ± 1.7	33.5 ± 2.5	33.3 ± 2.5	0.450

PRE premenopause, LPRE late premenopause, PERI perimenopause, POST postmenopause, BMI body mass index, V̇O_2_ volume of oxygen

*indicates significantly different to PRE, † indicates difference to PERI. ‡ indicates difference to POST

### Exercise intensity

There were no differences in exercise intensity between groups. There was a menopause stage effect (*p* < 0.001) but no interaction effect (*p* = 0.118) on %predicted VO_2peak_. Preliminary multiple comparisons suggested that intensity based on this metric was lower in PRE than PERI and POST in all conditions (*p* > 0.05) (Table 2).

**Table 2:** Mean and standard deviation for measures of exercise intensity across 30 min of steady-state exercise at 40%V̇O_2peak_ and 60%V̇O_2peak_ and 20 min at 80%V̇O_2peak_

Intensity (%V̇O_2_)	Pre (*n* = 35)	Peri (*n* = 19)	Post (*n* = 20)	*p column effect*	*p* *intensity x condition*
Power (W)					
40%	37 ± 22	28 ± 14	32 ± 18	0.204	0.408
60%	80 ± 26	82 ± 25	76 ± 22		
80%	118 ± 31	123 ± 23	107 ± 22		
% V̇O_2_R (%)					
40%	50 ± 7	50 ± 7	49 ± 8	0.724	0.365
60%	69 ± 5	69 ± 6	70 ± 5		
80%	90 ± 6	92 ± 8	91 ± 5		
% V̇O_2peak_ (%)					
40%	43 ± 6	43 ± 4	42 ± 5	0.255	0.375
60%	59 ± 4	59 ± 4	61 ± 3		
80%	77 ± 4	80 ± 6	79 ± 4		
V̇O2 (mL kg^−1^ min^−1^)					
40%	16.3 ± 4.0	16.1 ± 2.9	14.7 ± 3.0	0.194	0.450
60%	23.4 ± 5.2	22.0 ± 3.7	21.3 ± 3.5		
80%	30.5 ± 6.1	29.5 ± 4.9	27.7 ± 4.9		
Rating of perceived exertion					
40%	8 ± 1	8 ± 1	9 ± 2	0.476	0.563
60%	11 ± 2	11 ± 2	11 ± 2		
80%	14 ± 2	14 ± 2	14 ± 2		
Heart rate (b min^−1^)					
40%	103 ± 14	98 ± 16	93 ± 14	**0.017**	0.570
60%	127 ± 20	118 ± 15	115 ± 17		
80%	150 ± 22	140 ± 21	136 ± 21		
% Predicted V̇O_2max_					
40%	49 ± 8	58 ± 9	58 ± 9	** < 0.001**	0.118
60%	68 ± 13	80 ± 12	84 ± 12		
80%	89 ± 15	106 ± 14	109 ± 15		

PRE premenopause, LPRE late premenopause, PERI perimenopause, POST postmenopause, VO_2_R Volume of oxygen reserve,

*indicates significantly different to PRE, † indicates difference to PERI. ‡ indicates difference to POST. *p* indicates significance of one-way ANOVA

### Metabolic and ventilatory responses

There were no differences across groups in any of the metabolic variables. A significant main effect of hormonal status was observed for energy expenditure (*p* = 0.039), but with no significant interaction effect (*p* = 0.689). Exploratory pairwise comparisons demonstrate a lower energy expenditure in postmenopausal participants at 40% VO₂_peak_ compared to premenopausal (*p* < 0.05) (Tables 3, 4).

**Table 3:** Mean and standard deviation for energy expenditure, fat oxidation, and carbohydrate oxidation rates across 30 min of steady-state exercise at 40%V̇O_2peak_ and 60%V̇O_2peak_ and 20 min at 80%V̇O_2peak_

Intensity (%V̇O_2peak_)	Pre (*n* = 35)	Peri (*n* = 19)	Post (*n* = 20)	*p* column effect	*p* intensity x condition
Energy expenditure (kcal min^−1^)					
40%	5.6 ± 0.9	5.5 ± 0.7	4.9 ± 0.9		
60%	7.8 ± 1.3	7.8 ± 1.0	7.2 ± 0.9	**0.039**	0.689
80%	10.4 ± 1.7	10.3 ± 1.3	9.4 ± 1.5		
Fat oxidation (g min^−1^)					
40%	0.24 ± 0.08	0.30 ± 0.10	0.21 ± 0.10	0.091	0.684
60%	0.24 ± 0.14	0.25 ± 0.12	0.21 ± 0.11		
80%	0.13 ± 0.14	0.14 ± 0.15	0.09 ± 0.11		
Carbohydrate oxidation (g min^−1^)					
40%	0.83 ± 0.31	0.66 ± 0.21	0.75 ± 0.28	0.355	0.716
60%	1.37 ± 0.47	1.30 ± 0.36	1.30 ± 0.30		
80%	2.43 ± 0.87	2.23 ± 0.42	2.19 ± 0.59		
Respiratory quotient					
40%	0.87 ± 0.05	0.85 ± 0.05	0.88 ± 0.06	0.289	0.756
60%	0.91 ± 0.06	0.91 ± 0.05	0.91 ± 0.04		
80%	0.98 ± 0.07	0.97 ± 0.05	0.98 ± 0.05		
Energy expenditure (kcal min^−1^ kgFFM)					
40%	0.11 ± 0.02	0.11 ± 0.01	0.12 ± 0.02	0.901	0.743
60%	0.16 ± 0.03	0.15 ± 0.02	0.15 ± 0.02		
80%	0.20 ± 0.04	0.20 ± 0.02	0.20 ± 0.03		
Fat oxidation (g min^−1^ kgFFM)					
40%	0.005 ± 0.002	0.006 ± 0.002	0.004 ± 0.002	0.358	0.761
60%	0.005 ± 0.003	0.005 ± 0.002	0.004 ± 0.002		
80%	0.003 ± 0.003	0.003 ± 0.003	0.002 ± 0.002		

*indicates significance at an alpha level of 0.05

**Table 4:** Mean and standard deviation for ventilatory variables across 30 min of steady-state exercise at 40%V̇O_2peak_ and 60%V̇O_2peak_ and 20 min at 80%V̇O_2peak_

Intensity (%V̇O_2peak_)	Pre (*n* = 35)	Peri (*n* = 19)	Post (*n* = 20)	*p* column effect	*p* intensity x condition
Respiratory frequency (breaths min^−1^)					
40%	24.4 ± 3.2	22.4 ± 3.2	21.8 ± 3.8	0.076	0.509
60%	27.5 ± 3.8	25.6 ± 3.2	26.2 ± 3.9		
80%	32.8 ± 6.9	30.3 ± 4.6	31.7 ± 5.3		
Tidal volume (L)					
40%	1.2 ± 0.2	1.3 ± 0.2	1.2 ± 0.2	0.349	0.585
60%	1.6 ± 0.2	1.6 ± 0.2	1.5 ± 0.2		
80%	1.9 ± 0.3	1.9 ± 0.2	1.8 ± 0.2		
Minute ventilation (L min^−1^)					
40%	29.8 ± 5.1	28.6 ± 3.9	26.3 ± 4.6	0.118	0.895
60%	42.3 ± 8.0	40.3 ± 6.1	38.6 ± 4.9		
80%	60.9 ± 15.0	57.4 ± 9.5	55.9 ± 9.4		
Ventilatory equivalents (V̇E/V̇CO_2_)					
40%	28.5 ± 1.8	27.2 ± 6.4	28.4 ± 2.1	0.722	0.195
60%	27.9 ± 2.5	26.3 ± 6.0	27.9 ± 2.2		
80%	38.7 ± 2.9	27.8 ± 2.8	29.3 ± 2.5		
Ventilatory equivalents (V̇E/V̇O_2_)					
40%	24.8 ± 1.8	23.0 ± 5.4	24.9 ± 2.0	0.210	0.081
60%	25.5 ± 2.7	23.8 ± 5.6	25.5 ± 2.3		
80%	28.1 ± 3.8	26.9 ± 2.9	28.7 ± 3.2		
P_ET_CO_2_					
40%	38.0 ± 2.3	38.2 ± 2.3	38.6 ± 2.6	0.469	0.082
60%	39.1 ± 2.8	39.8 ± 2.8	39.3 ± 2.8		
80%	38.0 ± 3.5	39.3 ± 3.6	36.9 ± 2.7		

### Effect of hormone therapy and hormonal contraceptives

The hormone formulations of HT and HC users are available in supplementary materials (S1). POST NM had lower oestrogen than POST HT, PERI NM, PERI HC, and PRE NM (*p* < 0.05). There were no significant differences evidenced in multiple comparisons between groups in body composition. PERI HC had lower MET minutes per week than PRE NM (*p* = 0.039).

PRE HC and PRE NM were significantly than PERI HC, PERI NM, POST HT, and POST NM (*p* > 0.001). PERI HC and PERI NM were younger than POST HT (*p* > 0.05) (Table 5).

**Table 5:** Mean and standard deviation for participant characteristics when divided based on HC and HT use

	PRE HCa *n* = 14	PRE NM b *n* = 21	PERI HC c *n* = 10	PERI NMd *n* = 9	POST HT e *n* = 11	POST NMf *n* = 9	*p* value
Oestradiol (pg ml)	24.7 ± 27.6	32.0 ± 35.5^f^	49.1 ± 65.8^f^	60.0 ± 68.7^f^	29.9 ± 24.0^f^	4.01 ± 2.6^bcde^	0.002
Progesterone (ng ml)	0.5 ± 0.4	1.1 ± 1.7	1.1 ± 2.6	1.6 ± 2.9	1.0 ± 1.9	1.3 ± 3.3	0.147
Age (years)	31 ± 8^cdef^	33 ± 8^cdef^	48 ± 5^abe^	47 ± 5^abe^	55 ± 4^abcd^	55 ± 3^ab^	< 0.001
Height (cm)	168.0 ± 5.4	168.8 ± 6.5	170.0 ± 5.5	167.8 ± 5.4	166.0 ± 4.1	163.2 ± 4.1	0.041
Weight (kg)	66.9 ± 9.9	69.7 ± 9.8	71.0 ± 10.2	71.8 ± 10.5	69.4 ± 14.0	68.6 ± 8.9	0.667
BMI (kg m^−2)^	23.5 ± 3.7	24.4 ± 2.9	24.5 ± 2.9	25.6 ± 4.0	25.3 ± 5.8	25.7 ± 2.9	0.374
Body fat (%)	23.5 ± 9.8	26.5 ± 7.0	25.8 ± 6.9	27.5 ± 5.9	31.1 ± 10.4	31.9 ± 5.8	0.098
Body fat mass (kg)	16.7 ± 8.2	22.3 ± 5.7	19.0 ± 7.1	19.9 ± 7.0	22.5 ± 12.7	22.5 ± 6.7	0.262
Muscle mass (kg)	28.0 ± 1.8	28.28 ± 3.46	28.9 ± 2.9	28.1 ± 3.2	25.6 ± 2.0	25.3 ± 1.9	0.012
Fat-free mass (kg)	50.4 ± 3.0	51.0 ± 5.8	51.6 ± 5.6	50.8 ± 5.5	46.8 ± 3.5	46.1 ± 3.4	0.028
Metabolic equivalent minutes per week	2714 ± 889	3137 ± 1224^c^	1745 ± 609^b^	2346 ± 745	2275 ± 1341	1885 ± 719	0.031
V̇O_2peak_ (mL kg^−1^ min^−1^)	39.7 ± 9.0	39.33 ± 7.3	36.65 ± 6.6	37.7 ± 6.0	34.0 ± 8.0	36.2 ± 3.9	0.352

Hormone therapies and hormonal contraceptives had no effect on metabolic or ventilatory responses to exercise (Table 6).

**Table 6:** Mean and standard deviation for metabolic and cardiorespiratory variables across 30 min of steady-state exercise at 40%V̇O_2peak_ and 60%V̇O_2peak_ and 20 min at 80%V̇O_2peak_

	PRE HC *n* = 14	PRE NM *n* = 21	PERI HC *n* = 10	PERI NM *n* = 9	POST HT *n* = 11	POST NM *n* = 9	*p* column effect	*p* value Intensity x group
	*Pulmonary*							
	RF							
40%	25.4 ± 2.4	23.8 ± 3.2	23.3 ± 3.4	21.4 ± 2.2	21.3 ± 3.5	22.4 ± 3.8	0.079	0.669
60%	27.9 ± 3.5	27.1 ± 3.8	36.7 ± 3.0	24.4 ± 1.3	25.2 ± 3.8	27.4 ± 3.2		
80%	34.7 ± 7.2	31.6 ± 6.5	31.1 ± 5.3	29.3 ± 3.7	30.3 ± 5.6	33.3 ± 4.6		
	VT							
40%	1.2 ± 0.1	1.3 ± 0.2	1.3 ± 0.1	1.4 ± 0.1	1.3 ± 0.2	1.2 ± 0.1	0.303	0.910
60%	1.5 ± 0.2	1.6 ± 0.2	1.6 ± 0.2	16 ± 0.2	1.5 ± 0.3	1.5 ± 9,1		
80%	1.8 ± 0.2	1.9 ± 0.3	1.9 ± 0.2	2.0 ± 0.3	1.8 ± 0.3	1.7 ± 0.2		
	VE							
40%	29.8 ± 4.9	29.7 ± 5.0	28.9 ± 4.5	28.3 ± 3.0	26.6 ± 4.6	26.0 ± 4.5	0.485	0.978
60%	42.1 ± 8.3	42.3 ± 7.7	41.1 ± 5.9	39.3 ± 5.8	37.8 ± 5.0	39.5 ± 4.5		
80%	62.1 ± 15.7	60.0 ± 14.9	57.5 ± 9.6	57.3 ± 9.9	54.4 ± 9.8	57.7 ± 9.1		
	VE/VCO2							
40%	29.0 ± 2.1	28.2 ± 1.4	28.6 ± 2.5	28.8 ± 1.8	28.4 ± 1.8	28.4 ± 2.6	0.360	0.576
60%	28.7 ± 2.1	27.3 ± 2.5	27.7 ± 2.6	27.7 ± 1.3	27.7 ± 1.9	28.1 ± 2.5		
80%	29.8 ± 3.5	27.9 ± 2.1	28.0 ± 3.1	27.5 ± 2.6	29.5 ± 2.2	29.0 ± 2.9		
	VE/VO2							
40%	25.1 ± 1.7	24.7 ± 1.7	24.4 ± 2.2	24.0 ± 1.3	25.3 ± 1.5	24.5 ± 2.3	0.503	0.940
60%	25.8 ± 2.3	25.3 ± 2.8	25.6 ± 2.3	24.5 ± 2.0	25.7 ± 2.3	25.3 ± 2.4		
80%	28.9 ± 4.5	27.6 ± 3.3	27.1 ± 2.8	26.5 ± 3.1	28.9 ± 3.2	28.3 ± 3.4		
	PetCO2							
40%	37.1 ± 2.7	38.6 ± 1.8	38.1 ± 2.7	38.2 ± 2.0	38.5 ± 2.5	38.6 ± 2.9	0.286	0.466
60%	38.0 ± 2.5	39.8 ± 2.9	39.9 ± 3.7	39.8 ± 1.3	39.5 ± 2.6	39.1 ± 3.0		
80%	37.1 ± 4.3	39.3 ± 2.7	39.5 ± 4.3	40.3 ± 3.5	37.5 ± 2.6	37.9 ± 3.3		
	*Intensity*							
	HR							
40%	103 ± 14	102 ± 15	96 ± 15	102 ± 18	91 ± 12	95 ± 15	0.072	0.612
60%	126 ± 21	128 ± 21	117 ± 16	118 ± 14	107 ± 22	119 ± 14		
80%	152 ± 22	148 ± 22	142 ± 20	137 ± 22	130 ± 25	142 ± 13		
	RER							
40%	0.86 ± 0.04	0.87 ± 0.05	0.85 ± 0.05	0.84 ± 0.05	0.89 ± 0.07	0.86 ± 0.04	0.243	0.858
60%	0.90 ± 0.06	0.91 ± 0.06	0.93 ± 0.05	0.88 ± 0.04	0.93 ± 0.05	0.90 ± 0.03		
80%	0.96 ± 0.06	0.99 ± 0.07	0.97 ± 0.04	0.97 ± 0.07	0.98 ± 0.06	0.98 ± 0.05		
	VO_2_							
40%	16.9 ± 2.2	16.6 ± 2.9	16.0 ± 2.6	16.2 ± 3.4	14.8 ± 3.6	14.7 ± 2.2	0.465	0.823
60%	23.8 ± 5.9	23.2 ± 4.6	21.9 ± 3.3	22.1 ± 4.4	20.7 ± 4.2	22.1 ± 2.4		
80%	31.2 ± 7.2	30.0 ± 5.4	29.1 ± 4.9	29.9 ± 5.1	26.7 ± 6.1	28.8 ± 2.7		
	%VO_2max_							
40%	43 ± 5	42 ± 6	44 ± 4	43 ± 5	44 ± 7	41 ± 5	0.521	0.621
60%	60 ± 4	59 ± 3	60 ± 3	58 ± 4	61 ± 4	61 ± 2		
80%	78 ± 4	77 ± 3	80 ± 6	79 ± 5	79 ± 3	80 ± 4		
	%VO_2reserve_							
40%	51 ± 7	49 ± 7	51 ± 7	50 ± 6	51 ± 8	46 ± 6	0.248	0.707
60%	70 ± 3	67 ± 5	69 ± 6	68 ± 6	71 ± 5	70 ± 4		
80%	93 ± 6	88 ± 5	92 ± 10	91 ± 4	91 ± 5	91 ± 5		
	*Metabolic*							
	FATox							
40%	0.24 ± 0.07	0.24 ± 0.08	0.29 ± 0.12	0.31 ± 0.09	0.18 ± 0.12	0.23 ± 0.08	0.090	0.884
60%	0.26 ± 0.16	0.23 ± 0.13	0.20 ± 0.11	0.30 ± 0.10	0.17 ± 0.11	0.25 ± 0.08		
80%	0.16 ± 0.15	0.11 ± 0.13	0.12 ± 0.12	0.17 ± 0.18	0.09 ± 0.12	0.10 ± 0.11		
	CHOox							
40%	0.79 ± 0.30	0.85 ± 0.31	0.69 ± 0.21	0.63 ± 0.22	0.81 ± 0.33	0.68 ± 0.18	0.541	0.851
60%	1.29 ± 0.48	1.43 ± 0.46	1.44 ± 0.36	1.15 ± 0.31	1.32 ± 0.33	1.22 ± 0.23		
80%	2.27 ± 0.67	2.54 ± 0.98	2.28 ± 0.37	2.27 ± 0.56	2.13 ± 0.63	2.27 ± 0.55		
	EE							
40%	5.46 ± 0.85	5.61 ± 0.92	5.49 ± 0.78	5.45 ± 0.62	4.91 ± 0.94	4.90 ± 0.77	0.217	0.758
60%	7.69 ± 1.44	7.90 ± 1.18	7.64 ± 1.03	7.55 ± 0.99	6.99 ± 1.03	7.35 ± 0.71		
80%	10.22 ± 1.67	10.48 ± 1.68	10.25 ± 1.60	10.40 ± 1.10	9.10 ± 1.56	9.82 ± 1.26		

## Discussion

In this study, participants were all studied during low endogenous hormone states to assess the impact of chronically lower hormones against acutely lower hormones of the menstrual cycle. Whilst the participants presented similar hormone profiles, postmenopause and perimenopause are characterised by a reduced chronic exposure to oestrogen compared to premenopausal females (Mumford et al. 2012). This study reveals no differences in substrate oxidation or ventilatory responses to exercise across the menopausal transition or induced by hormone therapy or hormonal contraceptive use. However, menopausal status does influence resting and submaximal exercise energy expenditure which is dissipated when normalised to fat-free mass.

Despite studying all groups in conditions where endogenous hormones would be at the lowest point, hormonal variation is possible. It is expected that oestrogen be lower in the postmenopause group compared to the premenopause group; previous studies have reported oestrogen concentrations of 15.4 pg/ml in postmenopause and 97.0 pg/ml in premenopause (Pasqualini et al. 2019) and 33.4 pg/ml in the early follicular phase (Cramer et al. 2015). Early perimenopause can involve fluctuations of oestrogen up to higher levels than premenopause (Prior 1998) followed by a pronounced decline in oestrogen from 2 years prior to final menstrual bleed, this decline slows 2–6 years after final menstrual bleed, in postmenopause (Sowers et al. 2008). This high level of hormone variability was expected as menstrual cycle control is significantly more difficult in perimenopause. The present study included a perimenopausal group to help understand not only the difference between pre- and postmenopause, but also the transition period. This group are not frequently investigated in tightly controlled physiological studies due to the high levels of intra- and inter- individual physiological fluctuations across this stage of life, highlighting shortcomings in research studies in the effects of menopause.

This work supports the conclusions that sex hormone concentrations have a significant effect on EE (Day et al. 2005; Gavin et al. 2018) and that the menopausal transition may contribute to decreased EE (Lovejoy et al. 2008). Whilst it has recently been postulated that menopause has limited effect on resting EE aside from age (Karppinen et al. 2023), postmenopausal participants, but not perimenopausal participants, evidenced lower resting energy expenditure than premenopausal participants. This suggests that age is not the primary driver, since the effect was not continuous across groups. These findings indicate that menopause stage influences energy expenditure during submaximal exercise, but this is not impacted by exercise intensity. Preliminary analysis using multiple comparisons suggested a lower energy expenditure at low intensities in postmenopause compared to premenopause, in line with the findings of Abildgaard et al. (2013) at 50% V̇O_2peak_, which may suggest an intensity-dependent effect; however, this should be interpreted cautiously given the absence of an interaction effect. Previously, an exercise intensity-dependent effect has been demonstrated in the role of oestrogen and progesterone on exercise metabolism, whereby at high intensities of the exercise, the effect of hormones is negated due to the overriding cardiopulmonary and metabolic response to the increased energy demand (Hackney et al. 1994). All groups exhibited similar aerobic capacities; subsequently, our data imply that high physical fitness cannot mitigate EE declines despite previous reports (Duval et al. 2013; Gavin et al. 2018).

The reduced EE in postmenopause, however, can be attributed to deleterious changes in body composition. Whilst menopause may contribute to acceleration of age-related increases in fat mass (Toth et al. 2000; Greendale et al. 2019) in this study, all groups had similar body fat mass (kg) and body mass (kg). Yet, the POST group had a higher body fat percentage than PRE, accompanied with lower muscle mass and FFM than the PRE and PERI groups. When normalised to FFM, differences in EE dissipated. Hence the characteristic changes in body composition of the menopausal transition, such as increased visceral fat and reduced lean body mass, may be bi-directionally related to reduced EE stimulated by menopause (Marlatt et al. 2022). Despite a meta-analysis of ten studies demonstrating that oestrogen supplementation with hormone therapy could increase resting daily EE by up to 222 kcals (Weidlinger et al. 2023), HT and HC had no effect on energy expenditure. This corroborates the hypothesis that temporal changes in sex hormones alone do not exert effect on EE, but the effects of chronic losses are related to changes in body composition over time.

Postmenopausal participants evidenced lower resting V̇E than premenopausal participants without differences in VT and RF, similar to findings of MacNutt et al. (2012) and Slatkovska et al. (2006) in menstrual cycle differences, possibly relating to reduced overall sex hormone exposure and reduced metabolic demand. However, there were no differences in exercise ventilation. MacNutt et al. (2012) suggested that sex hormones may contribute to lowering of the ventilatory recruitment threshold which results in an increased ventilatory response to CO_2_ and higher resting V̇E. This has been evidenced in the luteal phase of the menstrual cycle compared to the follicular phase (Dombovy et al. 1987; Das 1998; MacNutt et al. 2012; Rattley et al. 2025). This is related primarily to progesterone which acts as a stimulant for respiration (Regensteiner et al. 1989; León-Velarde et al. 2001). Whilst there have been no reported differences in ventilation at rest between premenopausal females in the early follicular phase and postmenopausal females (Mercuro et al. 2006; Preston et al. 2009; Rael et al. 2021), a blunted central respiratory chemoreflex to increase ventilation in response to increasing partial pressure of CO_2_ has been previously demonstrated in postmenopausal females at rest (Preston et al. 2009) and in ovariectomised rats (Marques et al. 2015). This was further elucidated by Davenport et al. (2012) in which active postmenopausal participants, but not sedentary participants, demonstrated this blunted ventilatory response to increasing CO_2_, which the authors relate to mechanical capacity and chemoreceptor sensitivity. This study did not evidence differences in minute ventilation across the female lifecycle at any exercise intensity, suggesting that cardiopulmonary response to exercise overrides any hormonally regulated effect. However, it is important to note that all groups demonstrated similar progesterone concentrations, which may explain the lack of differences.

## Limitations

This study did not employ dietary control outside of avoiding food consumption in the 2 h prior to the exercise tests. As conclusions are specific to their contexts, this study sought to investigate female participants in general lifestyle conditions without dietary manipulations to enable greater generalisability of results. Comparisons were made between groups and not within groups, ensuring that individual dietary consistency within participants was also not essential. However, the use of food diaries could have helped assess dietary differences between menopause groups and their potential influence on the results. Further, a previously validated protocol for resting energy expenditure was not employed and this should be considered in the interpretation of resting data. Additionally, continuous V̇O_2_ monitoring allows study of consistent metabolic intensity by modulating workload. However, in some cases, participants were unable to achieve this V̇O_2_ consistently in high-intensity conditions, leading to an overall lower percentage of V̇O_2peak_ over the exercise bout. This method should be employed with caution in high-intensity exercise conditions. This work is limited by the array of hormonal contraceptives utilised in the sample, limiting subgroup analysis of exogenous hormone effects. There are limitations of grouping different types of hormonal contraceptives together under an umbrella term; hence, further research should seek to elucidate the impact of hormonal contraceptives on resting and exercise metabolism with optimal controls in place (Flood et al. 2024).

## Conclusion

This study supports the conclusion that sex hormones exert an effect on exercise energy expenditure that may be exercise-intensity dependent. However, there is a limited effect of menopause stage on substrate metabolism and ventilation likely as the increased physiological demand in response to exercise surpasses the hormone-regulated blunting of ventilation and metabolism.

## Supplementary Information

Below is the link to the electronic supplementary material.

Supplementary file1 (DOCX 17 KB)

## Data Availability

The data that support the findings of this study are available from the corresponding author, CR, upon reasonable request.
