# Supplementary material for: The effect of intensity on metabolic and ventilatory responses to steady-state exercise in women across the adult lifecycle
Source: Eur J Appl Physiol. 2025 Sep 19;126(2):1127–39. doi: 10.1007/s00421-025-05981-1 (PMC12948886; doi:10.1007/s00421-025-05981-1)
Supplement: Supplementary file 1 — Supplementary file1 (DOCX 17 KB) [file 421_2025_5981_MOESM1_ESM.docx]

**Supplementary material**

*Table S1: Exogenous hormone formulations for contraceptive or hormone replacement therapy users.*

|  | **Progestogen only pill formulation (n=4)** | **Monophasic combined pill formulation**  **(n=8)** | **Long-acting reversible contraceptives (LARC)**  **(n=11)** | **Hormone replacement therapy**  **(n=11)** |
| --- | --- | --- | --- | --- |
| **PRE** | 75μg desogestrel (Cerazette) - 3 users | 35μg ethinylestradiol and 250μg norgestimate (Cilique) (1^st^) - active  30μg ethinylestradiol and 150μg levonorgestrel (Rigevidon) (2^nd^) - active  30μg ethinylestradiol and 75μg  gestodene (Millinette)(3^rd^) - active  30μg ethinylestradiol and 3mg drospirenone (Yacella) (4^th^) – inactive  30μg ethinylestradiol and 150μg desogestrel (Gederal) (3^rd^) - active  30μg ethinylestradiol and 150μg levonorgestrel (Rigevidon) (2^nd^) - active  30μg ethinylestradiol and 3mg drospirenone (Yasmin) (4^th^) - active | ~20 µg/day levonorgestrel (Mirena coil) - 3 users  ~30-40 µg/day (Implanon) |  |
| **PERI** | 75μg desogestrel (Desogestrel) | 35μg ethinylestradiol and 250μg norgestimate (Cilique) (1^st^) - active | ~20 µg/day levonorgestrel (Mirena coil) - 7 users | 1.5mg oestradiol (Oestrogel)  0.75mg oestradiol (Oestrogel)  ~100 µg/day oestradiol (Evorel patch 6.4 mg) |
| **POST** |  |  |  | 3.2mg estradiol hemihydrate, 11.2mg norethisterone acetate (Evorel Conti Patch)  3.2mg estradiol hemihydrate, 11.2mg norethisterone acetate (Evorel Conti Patch)  2mg as estradiol hemihydraten and 1mg norethisterone acetate (Kliofem)  2.25mg oestradiol (Oestrogel x3)  100mg micronized progesterone (Utrogestan)  1mg estradiol hemihydrate) and 0.5mg norethisterone acetate (Kliovance)  100mg micronized progesterone (Utrogestan) and 0.75mg oestradiol (Oestrogel)  ~20 µg/day levonorgestrel (Mirena coil) – 2 users  2mg estradiol hemihydrate and 1mg norethisterone acetate (Elleste Duet Conti pill)  Patches (undislosed) |
